# Rad17 Translocates to Nucleolus upon UV Irradiation through Nucleolar Localization Signal in the Central Basic Domain

**DOI:** 10.3390/ijms232012300

**Published:** 2022-10-14

**Authors:** Yasunori Fukumoto, Masayoshi Ikeuchi, Yuji Nakayama, Yasumitsu Ogra

**Affiliations:** 1 Graduate School of Pharmaceutical Sciences, Chiba University, 1-8-1 Inohana, Chuo-ku, Chiba 260-8675, Japan; 2 Department of Biochemistry & Molecular Biology, Kyoto Pharmaceutical University, Kyoto 607-8414, Japan

**Keywords:** Rad17, DNA damage response, nucleolus, nucleolar localization, subnuclear localization

## Abstract

The nucleolus is a non-membranous structure in the nucleus and forms around ribosomal DNA repeats. It plays a major role in ribosomal biogenesis through the transcription of ribosomal DNA and regulates mRNA translation in response to cellular stress including DNA damage. Rad17 is one of the proteins that initiate and maintain the activation of the ATR pathway, one of the major DNA damage checkpoints. We have recently reported that the central basic domain of Rad17 contains a nuclear localization signal and that the nuclear translocation of Rad17 promotes its proteasomal degradation. Here, we show that the central basic domain contains the nucleolar localization signal as well as the nuclear localization signal. The nucleolar localization signal overlaps with the nuclear localization signal and is capable of transporting an exogenous protein into the nucleolus. Phosphomimetic mutations of the central basic domain inhibit nucleolar accumulation, suggesting that the post-translational modification sites regulate the nucleolar localization. Nucleolar accumulation of Rad17 is promoted by proteasome inhibition and UV irradiation. Our data show the nucleolar localization of Rad17 and suggest a possible role of Rad17 in the nucleolus upon UV irradiation.

## 1. Introduction

The nucleolus is a non-membranous structure in the nucleus and forms around ribosomal DNA (rDNA) repeats. It plays a major role in ribosomal biogenesis through the transcription of rDNA by RNA polymerase I and regulates mRNA translation in response to cellular stress including DNA damage [1]. DNA damage response inhibits RNA polymerase I transcription, and the inhibition requires the ATM pathway, one of the major DNA damage checkpoints [2]. Another major DNA damage checkpoint is the ATR pathway, which responds to various chemical forms of DNA damage [3]. The canonical ATR pathway is one of the major checkpoint reactions outside the nucleoli; however, a previous report has disclosed that the ATR pathway is activated in the nucleolus upon inhibition of RNA polymerase I transcription [4], indicating that the ATR pathway also monitors genomic DNA in the nucleolus. 

Rad17 is one of the proteins that initiate and maintain the activation of the ATR pathway. Rad17 loads the Rad9–Hus1–Rad1 complex (9–1–1 complex) onto damaged chromatin to activate ATR kinase activity. Rad17 also interacts with the Mre11-Rad50-NBS1 complex to activate ATM kinase [5]. The nuclear localization of endogenous Rad17 in non-irradiated [5,6,7] and irradiated [5,8] cells has been reported, and Rad17-S645 phosphorylation signal has been observed in the nucleus [9,10]. By contrast, the nucleolar localization of Rad17 has been poorly characterized. There is one report of the nucleolar staining of endogenous Rad17 [11]; however, the staining was accomplished with only one monoclonal antibody clone, and the possibility of nonspecific staining was not excluded. Other reports have indicated that Rad17 is primarily localized in the nucleoplasm but not in the nucleolus [12,13]. 

**Figure 1:**
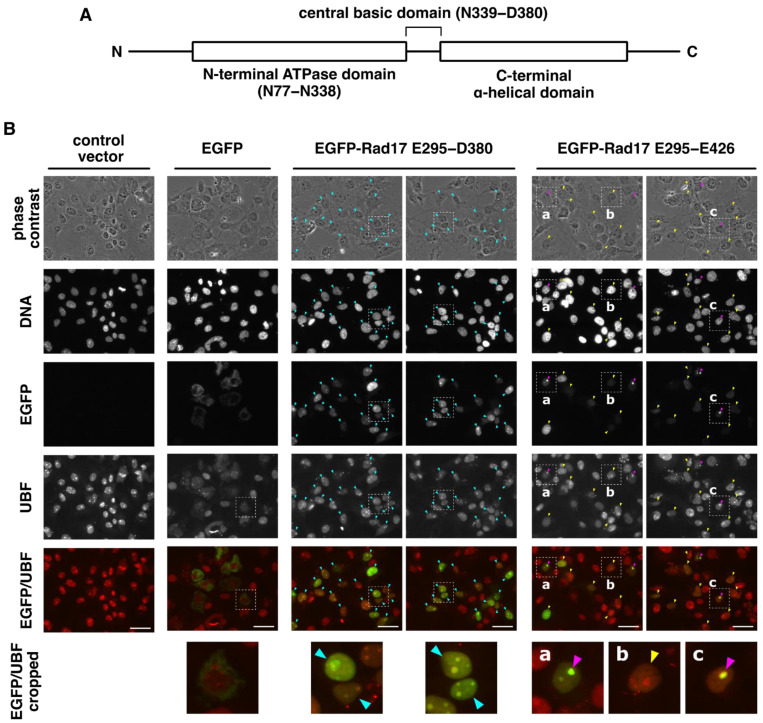
The central basic domain of Rad17 encodes a nucleolar localization signal. (**A**) The central basic domain resides between the N-terminal ATPase domain and the C-terminal α-helical domain. The domain structure of human Rad17 is shown (isoform 1. NCBI NP_579921.1. 670 amino acids). (**B**) EGFP fused with Rad17 E295–D380 and E295–E426 peptides was localized in the nucleus and predominantly accumulated in the nucleolus. COS-7 cells were transfected with plasmid vectors expressing EGFP fused with Rad17 E295–D380 or E295–E426 peptide. The cells were fixed and stained with anti-UBF antibody and Hoechst 33342. Arrowheads indicate cells in which the EGFP signal was localized in the following manners: cyan, in the nucleus and predominantly accumulated in the nucleolus; magenta, in the nucleolus; yellow, in both nucleus and nucleolus. Representative results of two independent experiments are shown. The ratios of cells with the magenta arrowhead to all EGFP-positive cells were 34% and 22% in each experiment. Approximately 100 cells were observed in each experiment. Scale bars are 40 μm.

Human Rad17 has a cluster of basic residues, N339–D380, which we named the central basic domain, and this domain is located between N-terminal ATPase and C-terminal α-helical domains (Figure 1A). We have recently reported that the central basic domain contains a nuclear localization signal and that the nuclear translocation of Rad17 promotes its proteasomal degradation. We have also identified tandem destruction boxes of Rad17 on its N-terminus as a set of canonical and noncanonical sequences [14]. The proteasomal degradation of Rad17 is mediated by an anaphase-promoting complex associated with Cdh1 [8], and the N-terminal destruction boxes of Rad17 interact with Cdh1 in vitro [14]. 

Here, we show that a central basic domain contains the nucleolar localization signal as well as the nuclear localization signal. The nucleolar localization signal overlaps with the nuclear localization signal and is capable of transporting an exogenous protein into the nucleolus. The basic K/R-rich motifs are the key determinants of protein localization in nuclear sub-compartments [15]. On the other hand, the positively charged K/R-rich arrays are nuclear PI(4,5)P2 recognition motifs that are essential for protein localization in nucleoli and nuclear speckles [16]. Indeed, the nucleolar PI(4,5)P2 is in the close proximity to crucial nucleolar constituent fibrillarin [17]. The K/R-rich motif containing proteins were identified in proteins, which are linked proteasomal degradation in an MS-based quantitative approach [16]. Phosphomimetic mutations of the central basic domain inhibit the nucleolar accumulation, suggesting that post-translational modifications regulate the nucleolar localization. Furthermore, UV irradiation promotes the nucleolar accumulation of Rad17, suggesting a nucleolar function of Rad17 in the DNA damage response. Our data show the nucleolar localization and the nucleolar localization signal of Rad17 and suggest a possible role of Rad17 in the nucleolus upon UV irradiation. 

## 2. Results and Discussion

### 2.1. The Central Basic Domain of Rad17 Encodes a Nucleolar Localization Signal 

In our recent study, we found that EGFP fused with Rad17 E295–D380 peptide showed exclusive nuclear localization [14]. In this study, we further characterized the central basic domain of Rad17 spanning N339–D380 (Figure 1A). Rad17 E295–D380 and E295–E426 peptides were fused with EGFP, and their localization was examined. EGFP-Rad17 E295–D380 was exclusively localized in the nucleus and predominantly accumulated in the nucleolus (Figure 1B, cyan arrowheads). Almost all of the EGFP-positive cells showed the same localization pattern. The nucleolus is made up of three components: the granular component, the dense fibrillar component, and the fibrillar center [18]. In the nucleolus, EGFP-Rad17 E295–D380 surrounds the UBF signal, a marker of the nucleolus fibrillar center. EGFP-Rad17 E295–E426 was also exclusively localized in the nucleus where it was localized solely in the nucleolus (Figure 1B, magenta arrowheads) or distributed in the nucleolus and the nucleoplasm (Figure 1B, yellow arrowheads). The ratios of cells with exclusive nucleolar localization, indicated by the magenta arrowheads, to EGFP-positive cells were 34% and 22% in each experiment. These data indicate that the central basic domain of Rad17 encodes a nucleolar localization signal as well as a nuclear localization signal. 

### 2.2. Rad17 K359–K363 Residues Encode the Nucleolar Localization Signal

**Figure 2:**
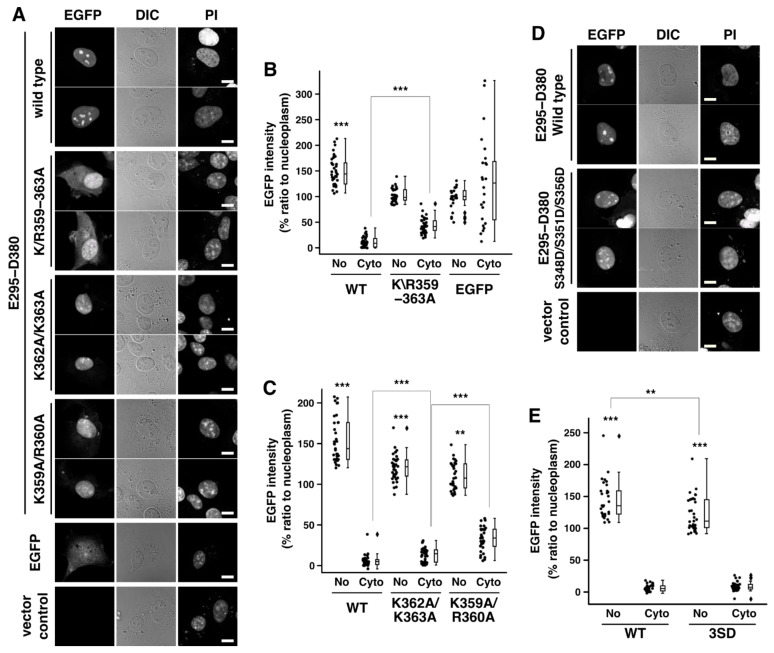
Rad17 K359–K363 residues encode the nucleolar localization signal. (**A**–**C**) Nucleolar accumulation of EGFP-Rad17 E295–D380 was abolished by K/R359–363A (A, B, S1A, S1B) and K359A/R360A (A, C, S1D, S1E) mutations. COS-1 cells were transfected with EGFP-Rad17 E295–D380 and fixed 48 h after transfection. DNA was stained with propidium iodide (PI). EGFP signal intensity was quantitated in the nucleolus, the nucleoplasm, and the cytoplasm, and the ratio to the nucleoplasm was calculated. (**D**,**E**) S348D/S351D/S356D (3SD) mutation abolished the nucleolar accumulation of EGFP-Rad17 E295–D380. The graph shows representative results of more than three independent experiments. Details of *p*-value calculation are shown in Figure S1. No, nucleolus. C, cytoplasm. DIC, differential inference contrast. ***, *p* < 0.001. **, *p* < 0.01. Scale bars are 10 μm.

In our previous work, we showed that Rad17 K359–K363 encoded a part of the nuclear localization signal and that K359A/R360A/R361A/K362A/K363A (K/R359–363A or 5KR) mutation abolished the nuclear localization of Rad17 [14]. Here, we examined the effect of K/R359–363A mutation on the nucleolar localization. EGFP-Rad17 E295–D380 having the wild-type sequence (WT) showed 149% accumulation in the nucleolus (referred to as No) relative to the nucleoplasm (Figure 2A,B and Figure S1A). The K/R359–363A mutant of this construct was deficient in the accumulation in the nucleolus and equally distributed in the nucleolus and the nucleoplasm. This mutation also increased the cytoplasmic localization (42%, Figure 2A,B and Figure S1A,B) relative to WT, as was shown recently [14]. EGFP alone was equally distributed in the nucleolus and the nucleoplasm, and no specific localization in the nucleoplasm or the cytoplasm was observed (Figure 2A,B and Figure S1A). The K359A/R360A mutant showed a slight accumulation in the nucleolus (110%) and a significant increase in cytoplasmic localization (33%, Figure 2A,C and Figure S1C,D). The K362A/K363A mutation had a milder effect; it decreased the nucleolar localization (120%) and slightly increased the cytoplasmic localization (13%, Figure 2A,C and Figure S1C,D). These findings indicate that K359 and R360 are central to the nuclear and nucleolar localization signals. We noted that in some cases, a nucleolar localization signal seemed to overlap with a nuclear localization signal [18]; however, we could not differentiate them. Our finding suggests that both signals overlapped in the central basic domain of Rad17. Together, the data indicate that Rad17 K359–K363 residues encode the nucleolar localization signal as well as the nuclear localization signal. 

### 2.3. Putative Phosphorylation Sites in the Central Basic Domain Regulate the Nucleolar Localization Signal of Rad17 

The phosphorylation of Rad17-S348, S351, and S356 residues was confirmed by mass spectrometric analyses and registered in PhosphoSitePlus (https://www.phosphosite.org). In our recent work, we noted that S348D/S351D/S356D mutation decreased the nuclear localization of flag-Rad17 full-length protein [14]. In the EGFP-Rad17 E295–D380 protein, the S348D/S351D/S356D mutation resulted in a decrease in nucleolar accumulation (121%, Figure 2D,E and Figure S1E,F) but did not affect the cytosolic intensity of the EGFP signal (Figure 2D,E and Figure S1F). These results indicate that the phosphorylation sites in the central basic domain regulate the nucleolar localization signal of Rad17. 

### 2.4. Proteasomal Degradation Negatively Regulates the Nucleolar Localization of Rad17 

**Figure 3:**
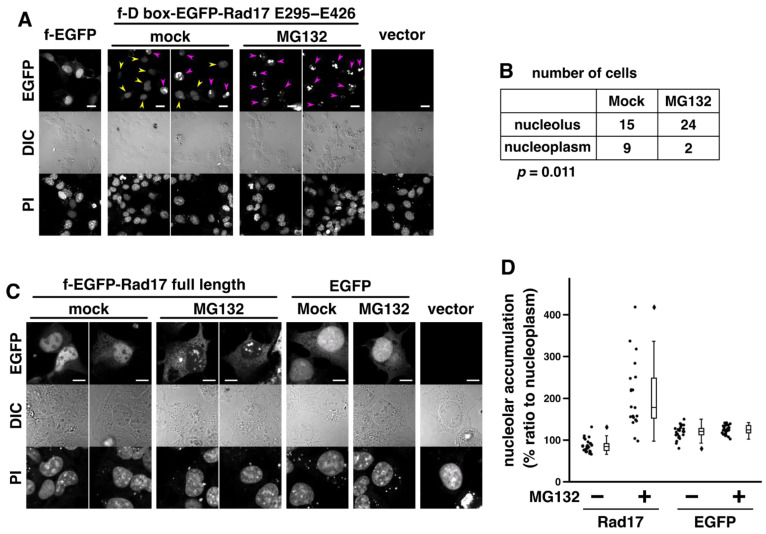
Inhibition of proteasomal degradation promotes nucleolar translocation of Rad17. (**A**,**C**) COS-1 cells were transfected with flag-D box (Rad17 H36–G66)-EGFP-Rad17 E295–E426 (**A**) or flag-EGFP-Rad17 full-length (**C**) and fixed 48 h after transfection. The cells were exposed to 20 μM MG132 for 7 h before fixation. Arrowheads indicate cells in which the EGFP signal was localized (magenta) or not localized (yellow) in the nucleolus. Scale bars are 20 μm (**A**) and 10 μm (**C**). (**B**) The signal intensity of flag-D box-EGFP-Rad17 E295–E426 was quantitated in the nucleolus and the nucleoplasm. The ratio of nucleolus to nucleoplasm was calculated, and cells with ratio higher than three were counted as nucleolar localization. Table shows the number of cells with nucleolar and nucleoplasmic localization of flag-D box-EGFP-Rad17 E295–E426 in two independent experiments. The *p*-value was calculated with the chi-square test. (**D**) The signal intensity of flag-EGFP-Rad17 full-length in the nucleolus and the nucleoplasm was quantitated. The ratio of nucleolus to nucleoplasm was calculated. The graph shows representative results of two independent experiments. Rad17 mock- and MG132-exposed groups showed significant difference with *p*-values of less than 0.001 in the Welch *t*-test. DIC, differential contrast. PI, propidium iodide.

In our recent work, we found that the nuclear translocation of Rad17 promotes the proteasomal degradation of Rad17 and that the degradation is mediated by N-terminal destruction boxes that interact with Cdh1 [14]. It was also shown that Cdh1 is localized in the nucleus but not in the nucleolus [19]. Here, we examined the relationship between proteasome and the nucleolar localization of Rad17. The Rad17 N-terminal destruction boxes (H36–G66) were fused with EGFP-Rad17 E295–E426 peptide, and the localization was examined. Again, E295–E426 peptide translocated the fused protein to the nucleolus (Figure 3A,B). We examined the effect of mutations in the Rad17 destruction boxes, K36A/P42A/R55A/L58A (KPRL); however, we obtained a marginal result in our preliminary experiments. We also compared protein amount and stability between flag-D box-EGFP-Rad17 E295–D380 and E295–E426; however, we observed small or no difference (data not shown). Then, we examined the effect of proteasome inhibition on the nucleolar localization. Exposure to proteasome inhibitor MG132 promoted the nucleolar accumulation of flag-D box-EGFP-Rad17 E295–E426 (Figure 3A,B). The MG132 exposure also promoted the nucleolar accumulation of flag-EGFP-Rad17 full-length protein (Figure 3C,D). These results suggest that proteasomal degradation negatively regulates the nucleolar localization of Rad17. Our result is consistent with a previous observation that proteasome inhibition induced the nucleolar accumulation of nuclear proteins including ATM [20]. 

Our current observations suggest that at least two mechanisms regulate the nucleolar localization of Rad17. The first mechanism is the negative regulation by phosphorylation of the central basic domain (Figure 2D,E). The second mechanism is the negative regulation by proteasomal degradation (Figure 3). We previously showed that the nuclear localization of Rad17 is dependent on the nucleotide binding of the Rad17 ATPase domain [21]. Because the nuclear and nucleolar localization signals overlapped in the central basic domain (Figure 2A–C), the Rad17 ATPase domain may also regulate the nucleolar localization signal as the third mechanism.

### 2.5. UV Irradiation Promotes the Nucleolar Localization of Rad17 

**Figure 4:**
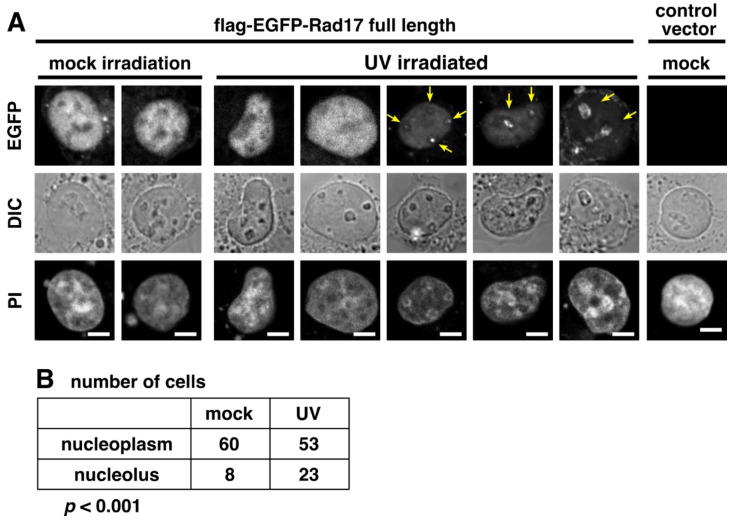
UV irradiation promotes nucleolar accumulation of flag-EGFP-Rad17 full-length protein. (**A**) COS-1 cells were transfected with flag-EGFP-Rad17 full-length. Forty-eight hours after transfection, the cells were exposed to 30 J/m^2^ UV-C and recovered for 3 h. The cells were fixed, and DNA was stained with propidium iodide (PI). Yellow arrows indicate nucleolar localization of the EGFP signal. DIC, differential contrast. Scale bars are 5 μm. (**B**) The number of cells with nucleoplasmic and nucleolar localization of flag-EGFP-Rad17 was counted in two independent experiments. The *p*-value was calculated with the chi-square test.

A previous report has demonstrated that Rad9B, a paralog of canonical Rad9 protein, translocates to the nucleolus upon UV irradiation [13]. Thus, we examined the effect of UV irradiation on the subnuclear localization of Rad17. The flag-EGFP-Rad17 full-length protein accumulated in the nucleolus of a subset of UV-irradiated cells (Figure 4A,B). Rad17 formed discrete foci in the nucleolus or distributed within the nucleolus. Because UV irradiation inhibits general transcription in the nucleus, one possible explanation may be that UV irradiation induces the nucleoplasmic depletion of Rad17 to result in the nucleolar accumulation. However, the inhibition of proteasomal degradation promoted the nucleolar accumulation of Rad17 (Figure 3), suggesting that the degradation or repression of Rad17 does not promote the nucleolar accumulation. These results indicate that UV irradiation induces the nucleolar translocation of Rad17. 

Rad9B interacts with Hus1, Rad1, and Rad17 but not with TopBP1, suggesting that it is not involved in the activation of ATR and the ATR-dependent DNA damage checkpoint [13]. To our knowledge, Rad9B, Hus1, and Rad1 do not have nucleolar localization signals. In *Schizosaccharomyces pombe*, Rad17 is required for the nuclear localization of Hus1 and Rad9 [22]. Rad17 may play a role in the translocation of Rad9B to the nucleolus. Upon DNA double-strand breaks, Rad17 directly interacts with NBS1 in the nucleoplasm [5], and NBS1 translocates to the nucleolus to inhibit rDNA transcription [23,24]. Rad17 and Rad9B may be involved in the nucleolar function of NBS1. The physiological function of Rad17 and Rad9B in the nucleolus is still a conundrum; however, our findings suggest the possibility that Rad17 and Rad9B cooperatively play a role upon UV irradiation. Further work will reveal the regulation and the nucleolar function of the Rad17 and Rad9B–Hus1–Rad1 complex.

## 3. Materials and Methods

### 3.1. Antibodies

The following antibodies were used: anti-UBF, F-9, Santa Cruz Biotechnology, sc-13125; rabbit anti-FLAG antibody, Medical & Biological Laboratories, PM020; anti-Hsc70, Santa Cruz Biotechnology, sc-7298; and anti-NPT2, Abcam, ab33595.

### 3.2. Plasmids

The amino acid residues of Rad17 were denoted according to isoform 1 (NCBI NP_579921.1). The pcDNA4 vectors encoding EGFP fused with Rad17 E295–D380 peptide (EGFP-Rad17 E295–D380) were described previously [14]. An EGFP fused with Rad17 E295–E426 peptide (EGFP-Rad17 E295–E426) was constructed in the same manner. The pcDNA3 vectors encoding flag-EGFP Rad17 full-length protein and flag-EGFP were described previously [14]. The pTwist CMV BetaGlobin WPRE Neo vectors encoding flag-D box-EGFP-Rad17 E295–D380 or E295–E426 peptide were synthesized by Twist Biosciences Inc. (San Francisco, CA, USA). The Rad17 H36–G66 sequence that contains tandem destruction boxes (D-box) was inserted between the flag tag and EGFP.

### 3.3. Fluorescence Microscopy 

We examined the co-localization of UBF and EGFP fused with the central basic domain of Rad17 as described previously [14,25]. COS-7 cells were transfected with 0.5 μg of pcDNA4/EGFP-Rad17 E295–D380 or E295–E426 peptide using Lipofectamine 2000 (Thermo Fisher Scientific, Waltham, MA, USA). The cells were fixed with 2% paraformaldehyde 24 h after transfection. The cells were stained with anti-UBF antibody and Alexa Fluor 555 Donkey anti-mouse IgG antibody (Thermo Fisher Scientific) in PBS (−)/3% BSA/0.1% saponin. DNA was stained with 1 μM Hoechst 33342. Fluorescence microscopic images were captured with an IX83 inverted fluorescence microscope (Olympus, Tokyo, Japan). 

We examined the subcellular and subnuclear localization of EGFP-Rad17 E295–D380, flag-EGFP Rad17 full-length protein, and flag-D box-EGFP-Rad17 E295–E426, as follows. COS-1 cells were transfected with 1.0 μg of plasmids using the acidified polyethylenimine [26]. The cells were fixed with 2% paraformaldehyde 48 h after transfection. To inhibit proteasomal degradation, the cells were exposed to 40 μM MG132 for 7 h before fixation. To examine the effect of UV irradiation, the cells were irradiated with 30 J/m^2^ of UV-C and allowed to recover for 3 h before fixation. The cells were treated with 200 μg/mL RNase A for 1 h and stained with 5 μg/mL propidium iodide for 30 min. The data were obtained with an LSM 700 or an LSM 5 Pa deconvolution microscope (Carl Zeiss, Jena, Germany). The average intensity of EGFP in the nucleolus, nucleoplasm, and cytoplasm was quantitated with ZEN 3.4 (blue edition) or Image J, and the average intensity ratio was calculated. The intensity of the nucleoplasm was used as 100% standard. The position of the nucleolus was determined on differential inference contrast or phase contrast. The dot and box–whisker plots were written with matplotlib v3.4.3 and seaborn v0.11.2. Whiskers represent the highest and lowest data, excluding outliers, and boxes represent 25%, 50%, and 75% percentiles. Student’s, Welch’s, or one-sample *t*-test was performed with the stat module of NumPy v.1.21.5 to calculate *p*-values. Extreme outliers that were larger than 75% quartile + 3 × (75% quartile − 25% quartile) were removed before plotting. 

## Supplementary Materials

The following supporting information can be downloaded at: https://www.mdpi.com/article/10.3390/ijms232012300/s1.

## Abbreviations

K/R359–363A or 5KR, K359A/R360A/R361A/K362A/K363A; KPRL, K36A/P42A/R55A/L58A; PI, propidium iodide; rDNA, ribosomal DNA; 3SD, S348D/S351D/S356D; 9–1–1 complex, Rad9–Hus1–Rad1 complex.
