# Supplementary material for: Rad17 Translocates to Nucleolus upon UV Irradiation through Nucleolar Localization Signal in the Central Basic Domain
Source: Int J Mol Sci. 2022 Oct 14;23(20):12300. doi: 10.3390/ijms232012300 (PMC9603387; doi:10.3390/ijms232012300)
Supplement: Supplementary file 1 [file ijms-23-12300-s001.zip › ijms-1954844-supplementary.pdf]

Supplemental Figure S1

A means ± standard deviation

	Nucleolus		Cytosol	
	mean ± SD	*p*-value	mean ± SD	*p*-value
WT	149 ± 28	< 0.001	10 ± 10	< 0.001
5KR	101 ± 12	NS	42 ± 15	< 0.001
EGFP	97 ± 21	NS	129 ± 87	NS

B *p*-values of *t*-test

	WT	5KR	EGFP	
WT		< 0.001	< 0.001	Cytosol
5KR	< 0.001		< 0.001	
EGFP	< 0.001	NS		
Nucleolus				

C means ± standard deviation

	Nucleolus		Cytosol	
	mean ± SD	*p*-value	mean ± SD	*p*-value
WT	153 ± 27	< 0.001	6 ± 7	< 0.001
K362A/ K363A	120 ± 16	< 0.001	13 ± 9	< 0.001
K359A/ R360A	110 ± 17	0.002	33 ± 15	< 0.001

D *p*-values of *t*-test

	WT	K362A/ K363A	K359A/ R360A	
WT		< 0.001	< 0.001	Cytosol
K362A/ K363A	< 0.001		< 0.001	
K359A/ R360A	< 0.001	0.011		
Nucleolus				

E means ± standard deviation

	Nucleolus		Cytosol	
	mean ± SD	*p*-value	mean ± SD	*p*-value
WT	149 ± 28	< 0.001	10 ± 10	< 0.001
3SD	121 ± 27	< 0.001	9 ± 7	< 0.001

F *p*-values of *t*-test (WT & 3SD)

Nucleolus	0.002
Cytosol	NS

Figure S1.

(A, C, E) Table shows the means ± standard deviation of the graphs in Figures 2B, 2C, and 2E. The *p*-values were calculated by one sample *t*-test. The signal intensity in the nucleoplasm was used as 100% control.

(B, D, F) Table shows the *p*-values of the Student's or Welch's *t*-test. WT and mutants were compared in terms of EGFP signal intensities in the nucleolus and the cytosol.
